# Trends in Inpatient Chemotherapy Hospitalizations, Cost and Mortality for Patients with Acute Leukemias and Myeloma

**DOI:** 10.1007/s44228-022-00003-9

**Published:** 2022-05-11

**Authors:** Kellen Gil, Saqib Abbasi, Kathan Mehta, Brian McClune, Douglas Sborov, Nausheen Ahmed, Al-Ola Abdallah, Siddhartha Ganguly, Joseph McGuirk, Leyla Shune, Ghulam Rehman Mohyuddin

**Affiliations:** 1 Department of Internal Medicine, University of Colorado School of Medicine, Aurora, USA; 2 Department of Hematological Malignancies and Cellular Therapeutics, Kansas University Medical Center, Kansas City, USA; 3 Department of Oncology, Kansas University Medical Center, Kansas City, USA; 4 Division of Hematology and Hematological Malignancies, University of Utah, Salt Lake City, USA

Patients with hematological malignancies such as acute myeloid leukemia (AML), acute lymphoid leukemia (ALL) and multiple myeloma (MM) have historically required lengthy inpatient hospitalizations for administration of intensive chemotherapy regimens. However, new treatment options and improved supportive measures have led to less treatment-related mortality and safer outpatient administration of treatment regimens [1, 2].

Using the National Inpatient Sample (NIS), we aimed to systematically analyze trends over time of hospitalizations for chemotherapy administration in patients with AML, ALL and MM. We hypothesized there would be a decrease in inpatient chemotherapy for acute leukemia and MM patients due to increased utilization of newer regimens delivered as an outpatient. We hypothesized there would be a decline in mortality for patients admitted for chemotherapy due to improvements in supportive care. Additionally, we aimed to explore inflation-adjusted costs for these hospitalizations.

The NIS is a database compiled by the Agency for Healthcare Research and Quality that contains data from more than 7 million annual hospitalizations including patient demographics, primary and secondary diagnoses, procedures, length of stay (LOS), and disposition. Each observation within the database comprises a unique hospitalization. Recurrent hospitalizations are recorded as distinct observations [3].

Weighted national estimates were obtained from the NIS using internal weighting from each state’s inpatient database. The two-sample *t* test was used to assess changes in inpatient mortality, length of stay, and total charges between the years 2002 and 2017. The years 2002 and 2017 were chosen for comparative analysis as they represented the first and last year of our study time period. We excluded hospitalizations for stem cell transplantation from our analysis.

Hospital charges were converted to costs using Healthcare Cost and Utilization Project cost-to-charge ratios [4]. Inflation adjustments for costs were done as per official US Department of Labor statistics. SPSS (Version 26, IBM Watson) was used for statistical calculations.

There were 136,839 hospitalizations from 2002 to 2017 for AML chemotherapy administration. The number of hospitalizations for AML chemotherapy administration decreased from 2008 onward. As a representative sample, there was a total of 3.74 hospitalizations per 100,000 population in 2008 compared to 1.72 per 100,000 population in 2017 (*p* = 0.003) (Fig. 1). Additionally, there was a decrease in inpatient mortality rates, from 6.9% in 2002 to 2.4% in 2017 (*p* < 0.0001). Mean LOS was 14.92 days in 2002 and 13.44 days in 2017 (*p* = 0.0054). Mean inflation-adjusted hospitalization costs increased from $83,890 in 2002 to $140,360 in 2017 (*p* < 0.001).

**Fig. 1:**
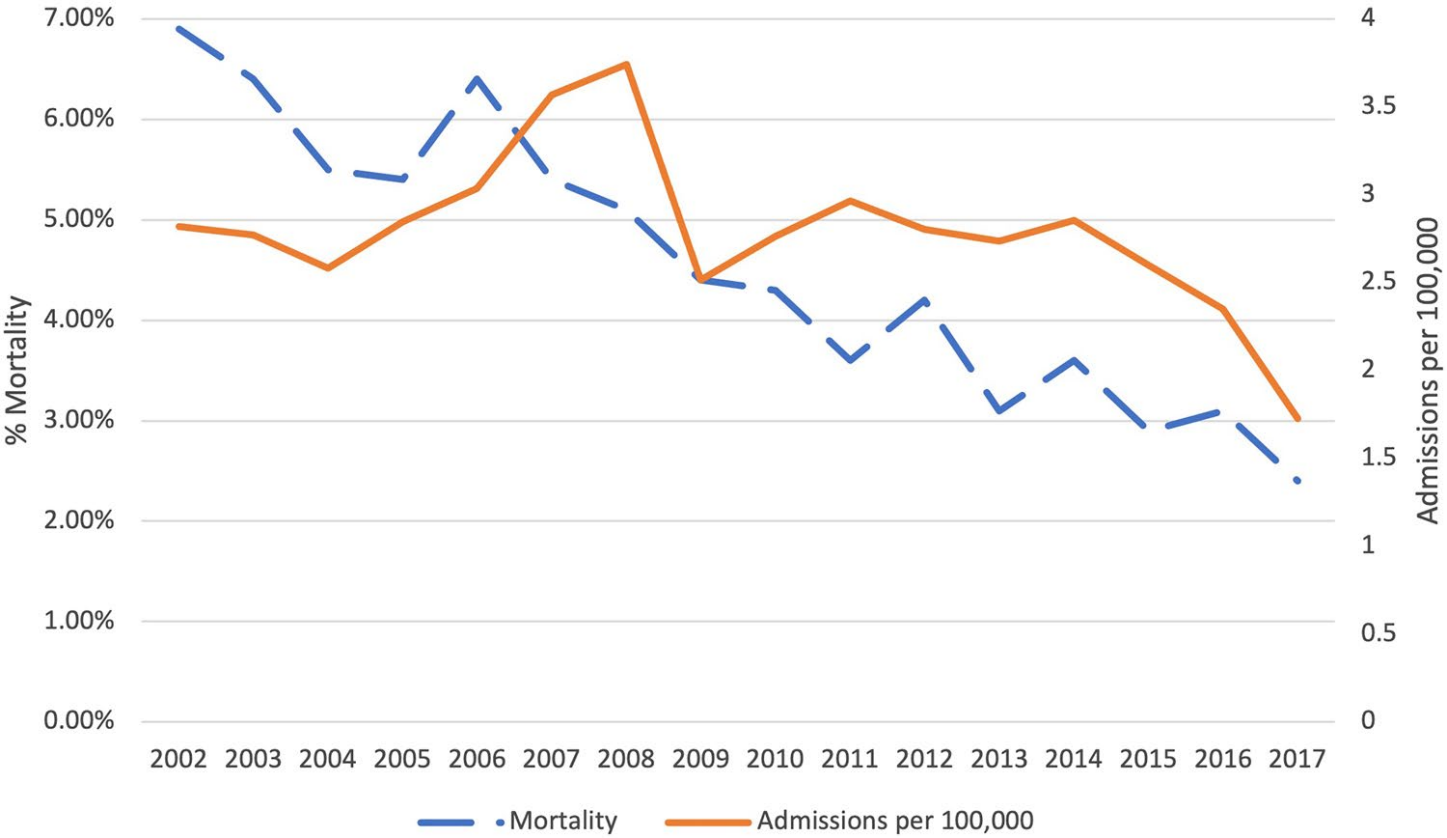
Trends in inpatient mortality and admission rates over time for acute myeloid leukemia in the United States

There were 82,730 hospitalizations from 2002 to 2017 for ALL chemotherapy administration. The number of hospitalizations for ALL chemotherapy administration did not change significantly over time. As a representative sample, there were 1.42 admissions per 100,000 population in 2002 and 1.83 admissions per 100,000 population in 2017 (*p* = 0.87) (Figure S1). There was a decrease in the inpatient mortality rate from 1.6% in 2002 to 0.8% in 2017 (*p* = 0.0007). Mean LOS did not change significantly during this time period at 7.70 days in 2002 to 7.63 days in 2017 (*p* = 0.06). Mean inflation-adjusted hospitalization costs increased from $49,283 in 2002 to $94,787 in 2017 (*p* < 0.0001).

There were 48,062 hospitalizations from 2002 to 2017 for MM chemotherapy administration. The number of hospitalizations for MM chemotherapy administration decreased significantly from 2.61 per 100,000 population in 2002 to 0.56 per 100,000 population in 2017 (*p* = 0.002) (Fig. 2). Inpatient mortality rates showed no significant change (0.7% in 2002 to 0.5% in 2017 (*p* = 0.15)). Mean LOS for these hospitalizations increased from 4.69 days in 2002 to 5.91 days in 2017 (*p* < 0.0001). Mean inflation-adjusted hospitalization costs increased from $18,781 in 2002 to $78,670 in 2017 (*p* < 0.0001).

**Fig. 2:**
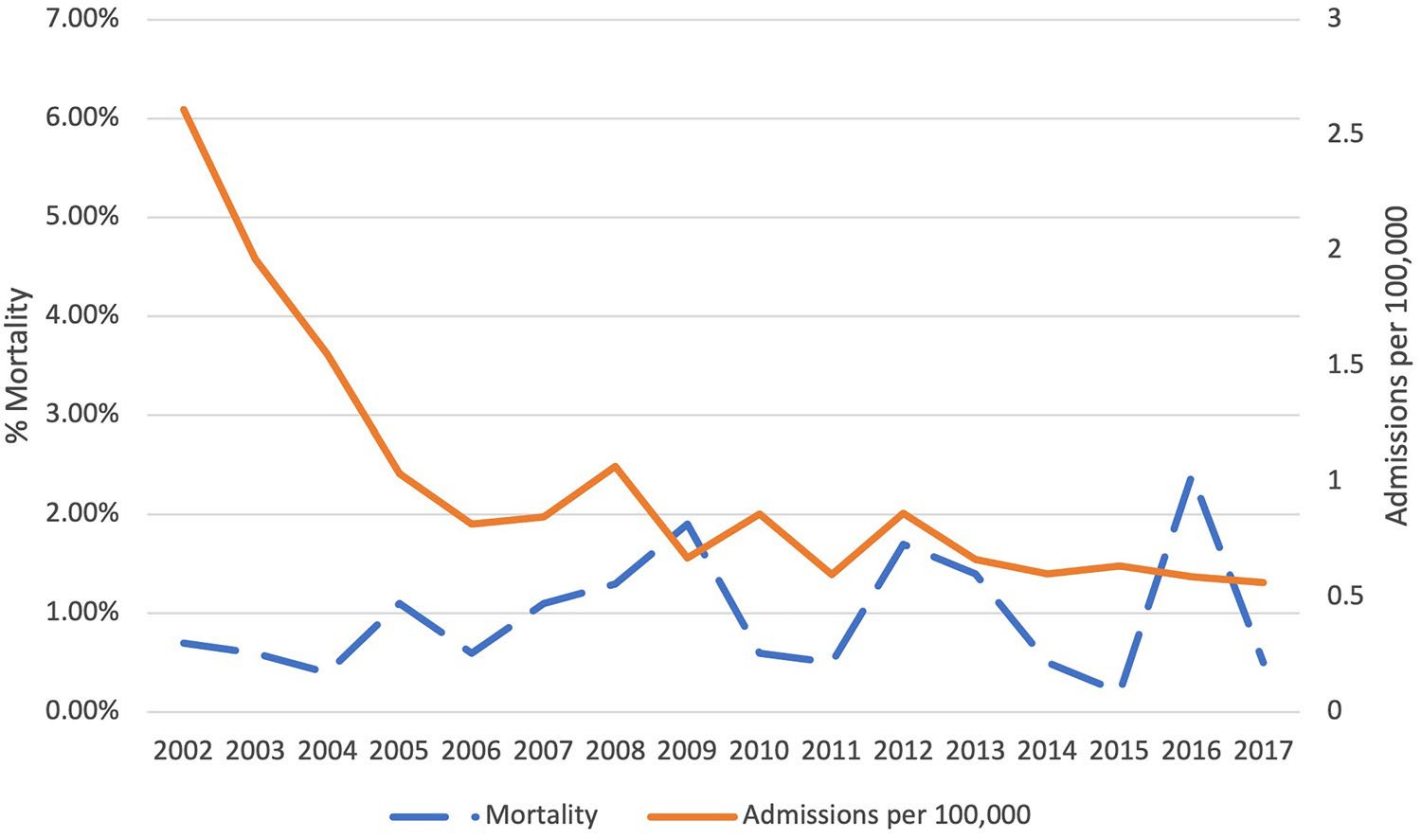
Trends in inpatient mortality and admission rates over time for multiple myeloma in the United States

In this study, we utilized national data spanning 15 years to analyze trends of inpatient chemotherapy administration, inpatient mortality, and hospitalization costs in adult patients with AML, ALL, and MM. We observed a steady decline over time in the number of hospitalizations for inpatient chemotherapy administration for patients with MM and AML, but not ALL. Inpatient mortality rates in AML and ALL patients admitted for chemotherapy administration decreased, however the inpatient mortality rate in MM patients did not significantly change. Furthermore, we found that inflation-adjusted hospitalization costs increased over time in patients with AML, ALL, and MM, despite the LOS remaining the same or only modestly increasing.

Our study reaffirms existing literature demonstrating an improvement in outcomes with chemotherapy administration for both AML and ALL [5, 6]. However, this is the first study to use a nationwide dataset capturing hospitalization-related data to establish trends in hospitalization volumes, outcomes, and costs for inpatient chemotherapy.

The reductions in inpatient mortality in AML and ALL are multifactorial with improvements in supportive care emerging from availability of efficacious antimicrobials [7], enhanced recognition of at-risk patients, and safer delivery of chemotherapeutic agents with recognition of their toxicities [8]. We found that the mortality rate of MM patients receiving inpatient chemotherapy has not significantly improved. We hypothesize this lack of improvement represents a shift towards a higher-acuity patient population receiving inpatient chemotherapy administration for MM, although the database limitations prevent us from specifically addressing this hypothesis.

Historically, LOS has been the largest determinant of the overall cost of hospitalization [9]. We show that despite the length of stay remaining relatively consistent over time, costs of hospitalizations have dramatically increased. Previous work shows that costs of hospitalizations across the United States (US) are going up, driven primarily by increased charges billed from the hospital for services delivered inpatient [10]. This is particularly relevant, as the chemotherapeutic agents used inpatient for these malignancies such as 7 + 3 for AML or cisplatin, doxorubicin, cyclophosphamide, and etoposide (PACE) for MM would not have been expected to get more expensive during our study time period. Newer, supportive care interventions such as contemporary antifungals may account for a small fraction of these costs, but do not explain the dramatic increase alone [11]. Newer agents for MM are primarily delivered outpatient, and contemporary inpatient chemotherapy agents for AML such as liposomal daunorubicin/cytarabine were not approved by the FDA until August 2017 [12]. The increase in costs cannot be attributed solely to increased complications during hospitalizations either, as we demonstrate that for acute leukemias the mortality rate has gone down significantly, and for MM has remained stable. There is an urgent need for further work in this regard, with particular focus on the exact mechanisms of what has driven these inpatient costs up, and what can be done to mitigate them.

There are several inherent limitations in all studies utilizing the NIS database. Data regarding leukemia or MM-specific patient characteristics is not available from the NIS database. This is compounded by lack of information provided by the NIS on comorbid factors which might increase costs and duration of hospital stays. Billing practices have also evolved since the transition to ICD-10-CM, therefore similar patients may have been coded differently before and after this transition. Furthermore, billing practices vary widely between institutions, and inaccurate documentation of ICD codes may affect the integrity of the information. Unfortunately admissions specifically for hospice cannot be tracked using this database, and disposition at discharge has also been removed from collected data and is not available for the years included in this study.

Despite those limitations, our results indicate there has been a steady decline in the number of hospitalizations for inpatient chemotherapy for patients with MM and AML in the US over time. We postulate this is due to increased utilization of effective outpatient agents for these diseases over time [13]. There has also been a steady decline in inpatient mortality for chemotherapy for AML and ALL. Hospitalization costs have gone up dramatically necessitating further work to elucidate factors driving inpatient healthcare expenditures.

## Supplementary Information

Below is the link to the electronic supplementary material.

Supplementary file1 (DOCX 28 kb)

## References

[1] Othus M, Kantarjian H, Petersdorf S (2014). Declining rates of treatment-related mortality in patients with newly diagnosed AML given ‘intense’ induction regimens: a report from SWOG and MD Anderson. Leukemia.

[2] Mabrey FL, Gardner KM, Shannon Dorcy K (2020). Outpatient intensive induction chemotherapy for acute myeloid leukemia and high-risk myelodysplastic syndrome. Blood Adv.

[3] Khera R, Krumholz HM (2017). With great power comes great responsibility: big data research from the national inpatient sample. Circ Cardiovasc Qual Outcomes.

[4] 2021 4/24. Healthcare Cost and Utilization Project. https://www.hcup-us.ahrq.gov/db/ccr/costtocharge.jsp. Accessed 24 Apr 2021.

[5] Hunger SP, Lu X, Devidas M (2012). Improved survival for children and adolescents with acute lymphoblastic leukemia between 1990 and 2005: a report from the children’s oncology group. J Clin Oncol.

[6] Percival ME, Tao L, Medeiros BC, Clarke CA (2015). Improvements in the early death rate among 9380 patients with acute myeloid leukemia after initial therapy: a SEER database analysis. Cancer.

[7] Freifeld AG, Bow EJ, Sepkowitz KA (2011). Clinical practice guideline for the use of antimicrobial agents in neutropenic patients with cancer: 2010 update by the Infectious Diseases Society of America. Clin Infect Dis.

[8] Walter RB, Othus M, Borthakur G (2011). Prediction of early death after induction therapy for newly diagnosed acute myeloid leukemia with pretreatment risk scores: a novel paradigm for treatment assignment. J Clin Oncol.

[9] Saxena SK, Ng TP, Yong D, Fong NP, Gerald K (2006). Total direct cost, length of hospital stay, institutional discharges and their determinants from rehabilitation settings in stroke patients. Acta Neurol Scand.

[10] Cooper Z, Craig S, Gaynor M, Harish NJ, Krumholz HM, Reenen JV (2019). Hospital prices grew substantially faster than physician prices for hospital-based care in 2007–14. Health Aff..

[11] Cornely OA, Maertens J, Winston DJ (2007). Posaconazole vs. fluconazole or itraconazole prophylaxis in patients with neutropenia. N Engl J Med.

[12] 2017 04/27. FDA approves first treatment for certain types of poor-prognosis acute myeloid leukemia. https://www.fda.gov/news-events/press-announcements/fda-approves-first-treatment-certain-types-poor-prognosis-acute-myeloid-leukemia. Accessed 27 Apr 2021.

[13] Rajkumar SV (2020). Multiple myeloma: 2020 update on diagnosis, risk-stratification and management. Am J Hematol.

