# Supplementary material for: Trends in Inpatient Chemotherapy Hospitalizations, Cost and Mortality for Patients with Acute Leukemias and Myeloma
Source: Clin Hematol Int. 2022 May 11;4(1-2):56–9. doi: 10.1007/s44228-022-00003-9 (PMC9358787; doi:10.1007/s44228-022-00003-9)

Figure S1: Trends over time in inpatient mortality and volume of hospitalizations in which chemotherapy was administered for acute lymphoid leukemia in the United States.


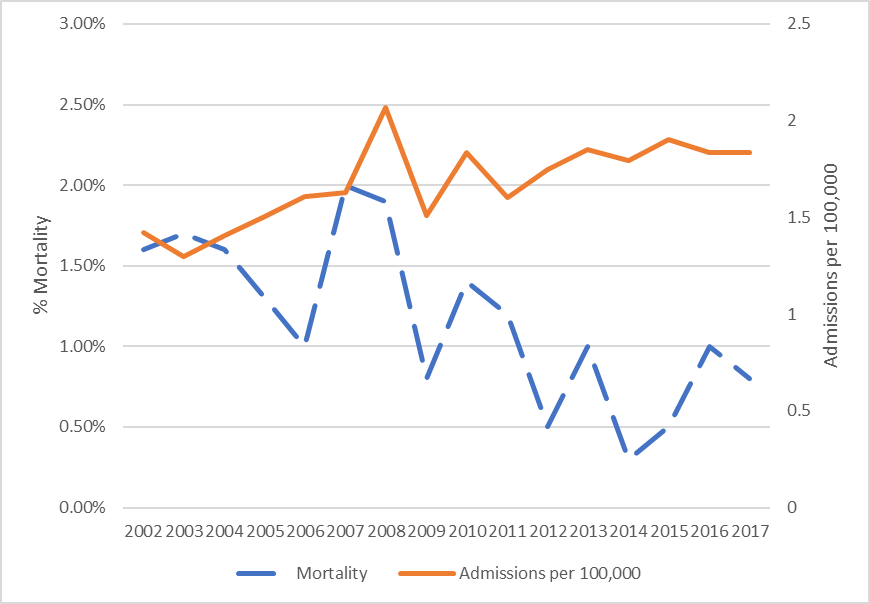

Supplement: Supplementary file 1 — Supplementary file1 (DOCX 28 kb) [file 44228_2022_3_MOESM1_ESM.docx]
